# Two-year clinical outcomes of a multicenter randomized controlled trial comparing two interspinous spacers for treatment of moderate lumbar spinal stenosis

**DOI:** 10.1186/1471-2474-15-221

**Published:** 2014-07-05

**Authors:** Vikas V Patel, Peter G Whang, Thomas R Haley, W Daniel Bradley, Pierce D Nunley, Larry E Miller, Jon E Block, Fred H Geisler

**Affiliations:** 1 University of Colorado Hospital, Denver, CO, USA; 2 Yale Orthopaedics/Spine Service, New Haven, CT, USA; 3 Performance Spine and Sports Physicians, P.C., Pottstown, PA, USA; 4 Texas Back Institute, Denton, TX, USA; 5 Spine Institute of Louisiana, Shreveport, LA, USA; 6 Miller Scientific Consulting, Inc., Asheville, NC, USA; 7 The Jon Block Group, 2210 Jackson Street, Suite 401, San Francisco, CA 94115, USA; 8 The Chicago Back Institute at Swedish Covenant Hospital, Chicago, IL, USA

**Keywords:** Interspinous spacer, Lumbar spinal stenosis, Minimally invasive, Randomized controlled trial, Superion

## Abstract

**Background:**

Interspinous spacers are a minimally invasive surgical alternative for patients with lumbar spinal stenosis (LSS) unresponsive to conservative care. The purpose of this prospective, multicenter, randomized, controlled trial was to compare 2-year clinical outcomes in patients with moderate LSS treated with the Superion® (Experimental) or the X-Stop®, a FDA-approved interspinous spacer (Control).

**Methods:**

A total of 250 patients with moderate LSS unresponsive to conservative care were randomly allocated to treatment with the Experimental (n = 123) or Control (n = 127) interspinous spacer and followed through 2 years post-treatment. Complication data were available for all patients and patient-reported outcomes were available for 192 patients (101 Experimental, 91 Control) at 2 years.

**Results:**

Zurich Claudication Questionnaire (ZCQ) Symptom Severity and Physical Function scores improved 34% to 36% in both groups through 2 years (all p < 0.001). Patient Satisfaction scores at 2 years were 1.8 ± 0.9 with Experimental and 1.6 ± 0.8 with Control. Axial pain decreased from 59 ± 26 mm at baseline to 21 ± 26 mm at 2 years with Experimental and from 55 ± 26 mm to 21 ± 25 mm with Control (both p < 0.001). Extremity pain decreased from 67 ± 24 mm to 14 ± 22 mm at 2 years with Experimental and from 63 ± 24 mm to 18 ± 23 mm with Control (both p < 0.001). Back function assessed with the Oswestry Disability Index similarly improved with Experimental (37 ± 12% to 18 ± 16%) and Control (39 ± 12% to 20 ± 16%) (both p < 0.001). Freedom from reoperation at the index level was 84% for Experimental and 83% for Control (log-rank: p = 0.38) at 2 years.

**Conclusions:**

Both interspinous spacers effectively alleviated pain and improved back function to a similar degree through 2 years in patients with moderate LSS who were unresponsive to conservative care.

**Trial registration:**

NCT00692276.

## Background

Lumbar spinal stenosis (LSS) is a common degenerative condition affecting the elderly that is characterized by narrowing of the spinal canal, lateral recesses, and/or neuroforamina that causes encroachment of surrounding soft tissue on the thecal sac and exiting nerve roots [1]. The natural history of LSS includes disease progression that may cause symptomatic neurogenic claudication, including pain in the buttocks or legs that is often exacerbated by standing, ambulating, and trunk extension. One of the classic features of LSS is pain alleviation with sitting or by standing/walking in a slightly flexed lumbar position.

The long-term effectiveness of nonsurgical LSS treatments such as activity modification, physical therapy, anti-inflammatory drugs, and epidural steroid injections is limited since these modalities have no impact on the rate of disease progression nor do they directly modify the diameter of the spinal canal [2-4]. In fact, 4 in 10 patients treated with conservative measures ultimately require decompressive surgery within 10 years due to symptom recurrence [5]. However, the potential for relief of claudication symptoms must be carefully balanced against the risks of treatment failure and surgical complications, particularly in the elderly [6]. There is a distinct treatment gap for patients with LSS who have unsuccessfully exhausted conservative treatments but whose symptom severity does not justify undergoing invasive decompression surgery.

Interspinous spacers are promising minimally invasive treatment alternatives for patients with persistent symptoms of LSS. Interspinous spacers are delivered via small, minimally traumatic incisions and implanted between contiguous spinous processes of a stenotic lumbar segment, with the goal of limiting back extension at the symptomatic level and alleviating neurogenic claudication symptoms. The purpose of this prospective, multicenter, randomized, controlled trial was to compare 2-year outcomes in patients treated with an investigational interspinous spacer or a Food and Drug Administration (FDA)-approved interspinous spacer.

## Methods

### Ethics

This clinical trial was conducted in strict accordance with a predefined protocol that was approved by all researchers and the institutional review board at each respective site [see Additional file 1]. This research followed the recommendations of the Helsinki Declaration and each patient provided written, informed consent before any study-related procedures were performed. This trial was prospectively registered at ClinicalTrials.gov (NCT00692276).

### Subjects

Inclusion criteria for this trial included: (a) age ≥ 45 years, (b) persistent leg, buttock, or groin pain, with or without back pain, that was relieved by lumbar flexion, (c) persistently symptomatic with unsuccessful response to at least 6 months of conservative treatment, (d) diagnosis of moderate LSS, defined as 25% to 50% reduction in central canal, lateral recess, or foraminal diameter compared to adjacent levels, and radiographic evidence of thecal sac compression and/or nerve root impingement by either osseous or non-osseous elements, and/or hypertrophic facets with canal encroachment, (e) Zurich Claudication Questionnaire Physical Function score ≥ 2.0, (f) able to sit for 50 minutes without pain and to walk ≥ 50 feet, and (g) able to provide voluntary informed consent and to comply with the study procedures. Exclusion criteria included: (a) LSS at three or more levels, (b) concomitant surgical procedure required, (c) grade II or greater spondylolisthesis, (d) unremitting back pain in any spinal position, (e) significant lumbar instability, defined as ≥ 3 mm translation or ≥ 5° angulation, (f) active systemic disease that may affect the welfare of the patient, (g) vertebral osteoporosis or history of vertebral fracture, (h) body mass index ≥ 40 kg/m^2^, (i) previous lumbar spine surgery, (j) pregnant or lactating female, and (k) any disease or condition that, in the investigator’s opinion, may affect subject safety or confound trial outcomes.

### Pre-treatment procedures

Pre-treatment evaluations included a physical examination, medical history, and assessment for study eligibility based on the inclusion/exclusion criteria. Radiographic assessments included x-rays (standing A/P, lateral lumbar, flexion/extension lateral lumbar) and magnetic resonance imaging or computed tomography of the lumbar spine. Self-reported measures included the Zurich Claudication Questionnaire (ZCQ) [7], a 100 mm visual analogue scale for extremity and axial pain severity, and the Oswestry Disability Index (ODI) (version 2) [8].

### Devices

**Figure 1:**
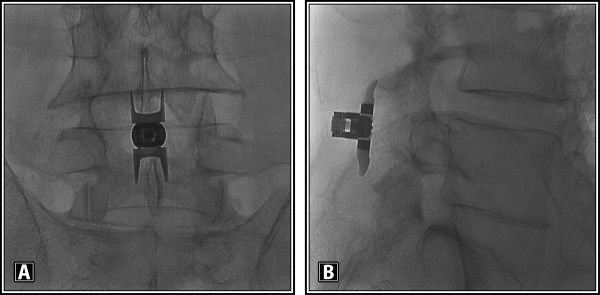
(A) A/P and (B) lateral radiographic image showing a properly placed Superion Interspinous Spacer.

Patients were randomized to treatment with the Superion Interspinous Spacer (VertiFlex, Inc., San Clemente, CA, USA) or a Control spacer (X-Stop Interspinous Process Decompression System; Medtronic, Inc., Sunnyvale, CA, USA). The Superion device (Figure 1A and 1B) is an investigational device that is composed of titanium 6AI-4 V ELI alloy, a material that conforms to ASTM standards for surgical implants and commonly used in a variety of orthopedic applications [9]. Five device sizes are available, ranging from 8 to 16 mm, with each size corresponding to the magnitude of desired distraction between the two spinous processes. This single-piece, self-expanding implant is delivered via minimally invasive access and deployed between the spinous processes of the involved vertebral levels. The Control spacer was approved for use in the United States by the FDA in November 2005 [10]. Procedural details have been described elsewhere [11]. Interspinous spacers were implanted at 1 (51%) or 2 (49%) levels, with a comparable distribution between groups.

### Follow-up

Subjects were followed through discharge and returned for visits at 6 weeks and 3, 6, 12, 18, and 24 months. Radiographic evaluations included standing A/P, lateral lumbar, and flexion/extension lateral lumbar x-rays. Postoperative care was prescribed according to individual subject needs and typically included medications, bracing, and/or physical therapy.

### Randomization and blinding

Treatment groups were randomly assigned using computer-generated codes. Site personnel accessed a web-based system to obtain treatment assignment before each subject was enrolled. Treatments were not concealed to investigators, outcome assessors, or trial participants.

### Data analysis

Data were analyzed using Predictive Analytics Software (v. 18, SPSS, Inc., Chicago, IL, USA). Continuous data were reported as mean ± SD and categorical data were reported as frequencies and percentages. Longitudinal changes in clinical outcomes were assessed with two-way (time x treatment) repeated measures analysis of variance. Clinical success was defined as a ≥20 mm improvement in pain scores [12,13] and a ≥15 percentage point improvement in ODI [12,14]. The Kaplan-Meier method and log-rank tests were used to analyze freedom from interspinous process fracture and reoperation at the index level.

## Results

### Subject characteristics

A total of 250 patients were randomized to Experimental (n = 123) or Control (n = 127) and followed for a minimum of 2 years. All patients were included in safety analyses while 192 (77%) patients had available 2-year patient-reported outcomes; the remaining patients withdrew or were lost to follow-up. Mean patient age was 67 years, 60% were male, and mean body mass index was 30 kg/m^2^. Grade I spondylolisthesis was identified in 34% of Experimental patients and 28% of Control patients. Baseline patient characteristics were comparable between the groups.

### Zurich Claudication Questionnaire

**Figure 2:**
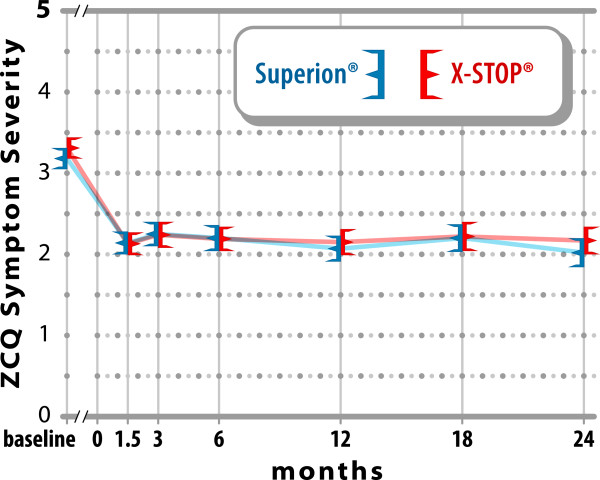
**Improvement in ZCQ symptom severity scores through 2 years post-treatment.** Values are mean ± 95% CI.

**Figure 3:**
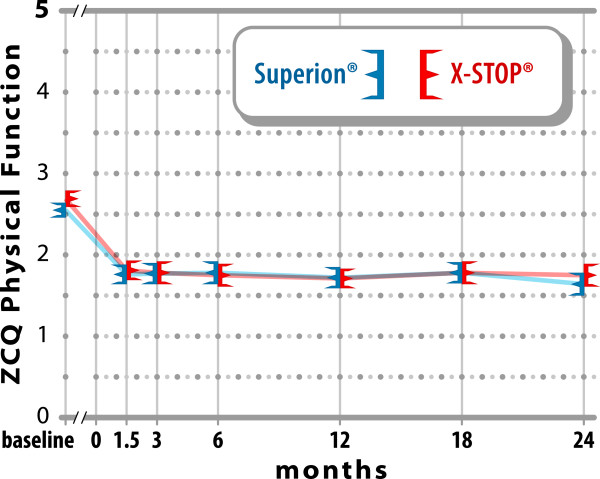
**Improvement in ZCQ physical function scores through 2 years post-treatment.** Values are mean ± 95% CI.

**Figure 4:**
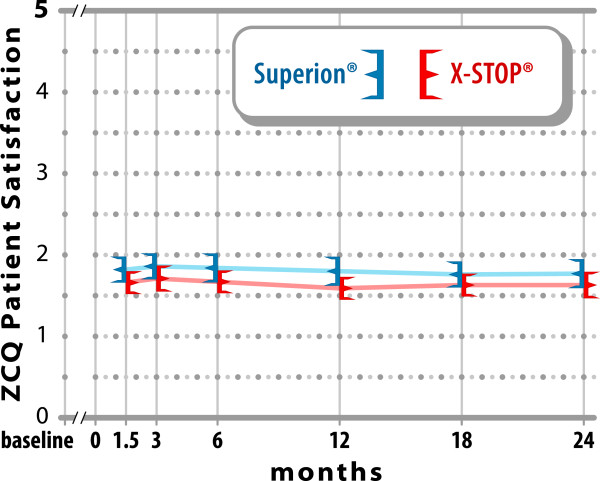
**ZCQ patient satisfaction scores through 2 years post-treatment.** Values are mean ± 95% CI.

ZCQ symptom severity scores improved 36% with Experimental and 34% with Control through 2 years (both p < 0.001; p = 0.60 between groups) (Figure 2). Similar changes were noted in ZCQ physical function with improvements of 36% with Experimental and 35% with Control (both p < 0.001; p = 0.54 between groups) (Figure 3). The mean ZCQ patient satisfaction score ranged from 1.6 to 1.9 in both groups at all follow-up visits (Figure 4).

### Axial pain severity

**Figure 5:**
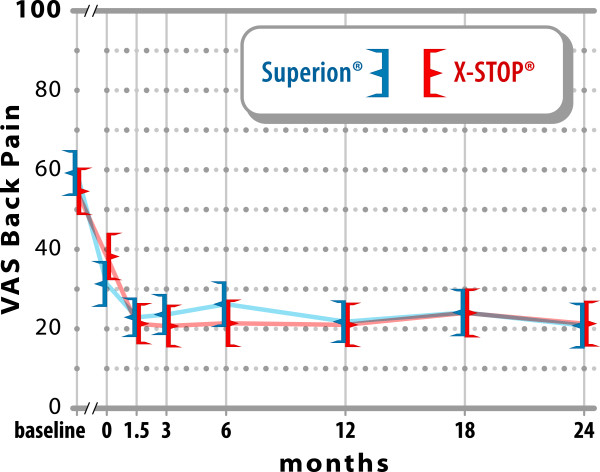
**Improvement in axial pain severity through 2 years post-treatment.** Values are mean ± 95% CI mm.

Axial pain decreased 64% (59 ± 26 mm to 21 ± 26 mm) at 2 years in the Experimental group and 62% (55 ± 26 mm to 21 ± 25 mm) with Control (both p < 0.001; p = 0.27 between groups) (Figure 5). At 2 years, 66% (67 of 101) of Experimental subjects and 62% (56 of 91) of Control subjects achieved axial pain clinical success. A strong positive relationship was noted between pre-treatment axial pain severity and magnitude of improvement following interspinous spacer treatment in both groups (Experimental, r = 0.67; Control, r = 0.62; both p < 0.001).

### Extremity pain severity

**Figure 6:**
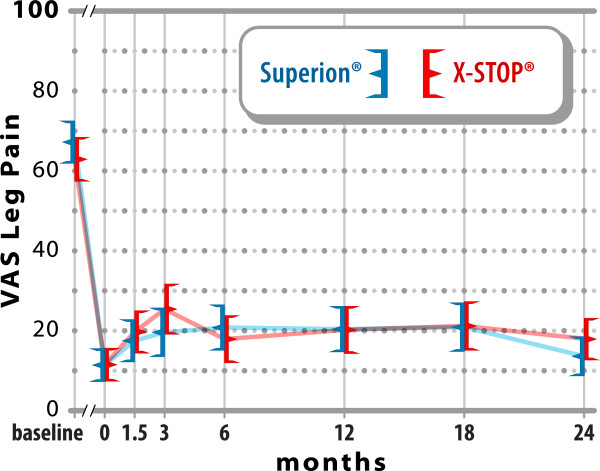
**Improvement in extremity pain severity through 2 years post-treatment.** Values are mean ± 95% CI mm.

Extremity pain decreased 79% (67 ± 24 mm to 14 ± 22 mm) at 2 years with Experimental and 71% (63 ± 24 mm to 18 ± 23 mm) with Control (both p < 0.001, p = 0.41 between groups) (Figure 6). At 2 years, 79% (80 of 101) of Experimental subjects and 75% (68 of 91) of Control subjects achieved extremity pain clinical success. A strong positive relationship was noted between pre-treatment extremity pain severity and magnitude of improvement following interspinous spacer treatment in both groups (Experimental, r = 0.66; Control, r = 0.72; both p < 0.001).

### Back-specific functional impairment

**Figure 7:**
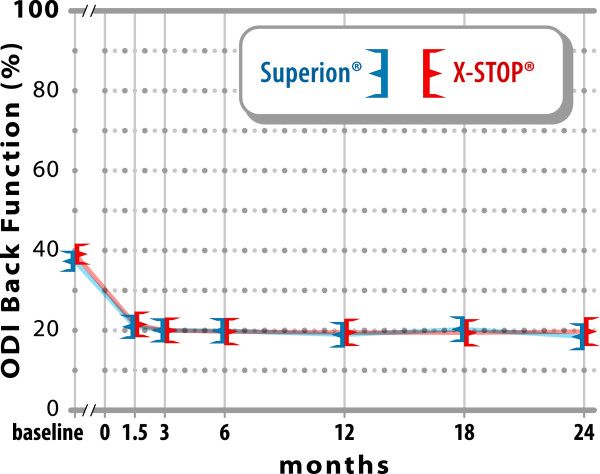
**Improvement in back function through 2 years post-treatment.** Values are mean ± 95% CI.

Back function improved 51% with Experimental (37 ± 12% to 18 ± 16%) vs. 49% with Control (39 ± 12% to 20 ± 16%) (both p < 0.001, p = 0.87 between groups) (Figure 7). At 2 years, 59% (60 of 101) of Experimental subjects and 60% (55 of 91) of Control subjects achieved back function clinical success. Weak relationships were noted between pre-treatment back function and magnitude of improvement following interspinous spacer treatment in either group (Experimental, r = 0.21, p = 0.04; Control, r = 0.44, p < 0.001).

### Complications

Postoperative deep wound infection was noted in 2 Control subjects, both successfully treated with incisional draining. The Kaplan-Meier estimate for freedom from a spinous process fracture was 93% for Experimental and 96% for Control (log-rank: p = 0.38) at 2 years. One Control patient underwent rhizotomy at 8 months post-implant. The Kaplan-Meier estimate for freedom from a reoperation at the index level was 84% for Experimental and 83% for Control (log-rank: p = 0.93) at 2 years. The timing of reoperations was 45% in the first 6 months, 21% between 6 and 12 months, and 33% in the second year. The types of reoperations performed in each group are shown in Table 1.

**Table 1:** Reoperations through 2 years

**Variable**	**Experimental**	**Control**
**Patients undergoing reoperation**	**15**	**18**
-Explant	12	14
-Decompression surgery	10	15
-Fusion	3	3
-Discectomy	1	2

Sum of procedures is greater than sum of patients due to multiple procedures.

### Subgroup analysis: Spondylolisthesis

Among patients with preoperative grade I spondylolisthesis, 2-year outcomes were consistently better in the Experimental group although these differences were not statistically significant due to the limited sample size in the subgroup analysis (Table 2).

**Table 2:** Clinical outcomes at 2 years in patients with preoperative grade I spondylolisthesis

**Variable**	**Experimental**	**Control**
Axial pain success, %^1^	64.7	47.8
Extremity pain success, %^1^	76.5	69.6
Back function clinical success, %^1^	61.8	56.5
Freedom from spinous process fracture, %^2^	100	94.1
Freedom from reoperation, %^2^	85.0	76.5

^1^Among patients who completed 2 years follow-up, preoperative grade I spondylolisthesis was identified in 34 (34%) of 101 in the Experimental group and 23 (25%) of 91 in the Control group.

^2^Among all patients included in the safety analyses, preoperative grade I spondylolisthesis was identified in 42 (34%) of 123 in the Experimental group and 36 (28%) of 127 in the Control group.

## Discussion

Patients with moderate LSS remain an underserved population with no acceptably safe and effective treatment options. Interspinous spacers represent a viable treatment alternative for these patients that bridge the gap between conservative care and decompression surgery. The 2-year clinical outcomes of this trial demonstrate clinically meaningful improvements in back function, back pain, and leg pain in patients treated with the Experimental and Control interspinous spacers.

The mid-term effectiveness of the Experimental device is comparable to data reported in two previous studies. Bini and colleagues [15] reported 1-year outcomes of 52 patients treated with the Experimental device for moderate LSS. In that study, axial pain severity improved 49% (p < 0.001), extremity pain severity improved 53% (p < 0.001), and back function improved 64% (p < 0.001). Shabat and colleagues [16] treated 53 patients with the Experimental device for moderate LSS and reported clinical outcomes through 2 years. Similarly, axial pain severity improved 54%, extremity pain severity improved 54%, back function improved 50%, ZCQ symptom severity improved 43%, ZCQ physical function improved 44%, and mean ZCQ patient satisfaction at 2 years was 1.9. Overall, the collective experience with the Experimental device to date suggests durable neurogenic claudication symptom amelioration. The effectiveness of the Experimental device in relieving symptoms of neurogenic claudication, the hallmark symptom of LSS, was particularly impressive with clinical success achieved in 8 in 10 patients in the current trial.

Data from the current study as well as from previous studies suggest that mid-term treatment effectiveness with interspinous spacers is comparable to that of open decompression surgery for moderate LSS [16-18]. The primary advantage of interspinous spacers is the minimally invasive approach, which is in stark contrast to the extensive resection of muscle, ligament, and bone that is typically required for traditional open decompression surgery. The specific procedural benefits of the Experimental procedure include a midline incision of only 1 cm, minimal disruption of the supraspinous ligament with preservation of the lamina and posterior ligamentous structures, short procedure time, and minimal blood loss. The lack of iatrogenic insult and associated complications combined with the postulated mechanism of action of immediate widening the spinal canal leads to rapid symptom improvement as evidenced by clinically meaningful neurogenic claudication symptom improvements at hospital discharge.

While the results of the present study are encouraging, the long-term durability of interspinous spacers is currently unknown and requires further study. Subjects in this trial will be followed through 5 years post-treatment. Although the clinical outcomes found in this study following treatment with the Experimental and Control spacers appear to be consistent with prior studies of open or minimally invasive decompression surgery, these decompression techniques were not directly compared with the Experimental device in this study, and thus comparisons of outcomes should be made with caution. With regard to patients with preoperative spondylolisthesis, long-term clinical outcomes with interspinous spacers were similar to those in patients without pre-existing spondylolisthesis. However, this study did not report the radiographic change in spondylolisthesis over time. Finally, we partly attribute the favorable results and low complication rates with both devices to the rigorous clinical and radiographic criteria employed in this clinical study. However, the generalizability of these outcomes to a real-world setting in patients with moderate LSS is unknown.

## Conclusions

Both interspinous spacers effectively improved neurogenic claudication symptoms through 2 years in patients with moderate LSS. The clinical improvements observed in this trial were similar between the Experimental and Control interspinous spacers.

## Competing interests

Drs. Miller and Block are consultants to VertiFlex, Inc. (San Clemente, CA, USA).

## Authors’ contributions

All authors were involved in study design and development of this manuscript. All authors read and approved the final manuscript.

## Pre-publication history

The pre-publication history for this paper can be accessed here:

http://www.biomedcentral.com/1471-2474/15/221/prepub

## Supplementary Material

Additional file 1 List of participating investigative sites. Click here for file
