# Supplementary material for: Two-year clinical outcomes of a multicenter randomized controlled trial comparing two interspinous spacers for treatment of moderate lumbar spinal stenosis
Source: BMC Musculoskelet Disord. 2014 Jul 5;15:221. doi: 10.1186/1471-2474-15-221 (PMC4109165; doi:10.1186/1471-2474-15-221)
Supplement: Additional file 1 — List of participating investigative sites. [file 1471-2474-15-221-S1.docx]

**Additional file 1. List of participating investigative sites.**

| **IRB Site Approved Address** |
| --- |
| Spine Institute of Louisiana  1500 Line Avenue Suite 200  Shreveport, LA 71101 |
| Specialist Hospital of Shreveport  1500 Line Avenue, Suite 206  Shreveport, LA 71101 |
| Orange County Neurosurgical Associates  23961 Calle de la Magdalena, Suite 504  Laguna Hills, CA 92563 |
| Saddleback Memorial Medical Center  24451 Health Center Drive  Laguna Hills, CA 92563 |
| George Washington University Medical Facility Associates  2150 Pennsylvania Avenue, NW Suite 7-416  Washington, DC 20037 |
| Anschutz Outpatient Clinic  1635 North Ursula Street  MS F722, Box 6510  Aurora, Colorado 80045 |
| University of Colorado Hospital  Anschutz Medical Campus  12605 East 16^th^ Avenue  Aurora, CO 80045 |
| Orthopedic Associates of Greater Lehigh Valley  3735 Easton-Nazareth Highway, Suite 101  Easton, PA 18045 |
| Warren Hospital  185 Roseberry Street  Phillipsburg, NJ 08865 |
| Sports Medicine North  One Orthopedics Drive, 2^nd^ Floor  Peabody, Massachusetts 01960 |
| Orthopedic Surgical Center of the North Shore  One Orthopedics Drive  Peabody, Massachusetts, 01960 |
| Upstate Bone and Joint Center  6620 Fly Road, Suite 200  East Syracuse, NY 13057 |
| SUNY University of New York  750 East Adams Street  Syracuse, NY 13210-2375 |
| Upstate Orthopedics Ambulatory Surgery Center  6620 Fly Road, Suite 300  Syracuse, NY 13057 |
| Triangle Orthopedics Associates, PA  120 William Penn Plaza  Durham, NC 27704 |
| Granville Medical Center  1010 College Street  Oxford, NC 27565 |
| North Carolina Specialty Hospital  3916 Ben Franklin Blvd  Durham, North Carolina 27704 |
| Triangle Orthopedic Associates, PA  103 Professional Park Drive  Oxford, NC 27565 |
| Performance Spine and Sports Physicians, P.C.  1603 East High Street, C Pottstown, PA 19464 |
| Pottstown Memorial Medical  1600 East High Street  Pottstown, PA 19464 |
| Texas Back Institute  2817 South Mayhill Road, Suite 100  Denton, Texas 76208 |
| Texas Back Institute, Plano  6020 West Parker Road, Suite 200  Plano, TX 75093 |
| Texas Back Institute, Rockwall  1005 West Ralph Hall Parkway, Suite 227  Rockwall, Texas 75032 |
| Texas Back Institute, Trophy Club  2800 West Highway 114, Suite 220  Trophy Club, Texas 76262 |
| Texas Back Institute, Mansfield  2800 East Broad Street, Suite 522  Mansfield, Texas 76063 |
| Texas Health Center for  Diagnostics and Surgery  6020 West Parker Road  Plano, TX 75093 |
| NeuroSpine Institute  74-B Centennial Loop, Suite 300  Eugene, Oregon 97401 |
| NeuroSpine Institute, LLC  74-B Centennial Loop, Suite 100  Eugene, Oregon 97401 |
| NorthWest NeuroSpine Institute  74-B Centennial Loop, Suite 200  Eugene, Oregon 97401 |
| Tower Orthopaedics and Sports Medicine  8670 Wilshire Boulevard, Suite 202  Beverly Hills, CA 90211 |
| Spine Cal  Pablo Pazmino, MD  2811 Wilshire Boulevard, Suite 800  Santa Monica, CA 90403 |
| Olympia Medical Center  5900 West Olympic Boulevard  Los Angeles, CA 90036 |
| Orthopedics Intl. Spine  901 Boren Avenue, Suite 800  Seattle, WA 98104 |
| Orthopedics Intl. Spine  901 Boren Avenue, Suite 900  Seattle, WA 98104 |
| Evergreen  12333 Northeast 130^th^ Lane Suite 400  Kirkland, WA 98034 |
| Evergreen Medical Center  12040 Northeast 128^th^ Street  Kirkland, WA 98034 |
| Orthopedics Intl. Ambulatory Surgery Center  600 Broadway, Suite 460  Seattle, WA 98122 |
| Greater Baltimore Neurosurgical Associates  Physicians Pavilion North  6535 N. Charles Street, Suite 600  Baltimore, MD 21214 |
| Greater Baltimore Medical Center  6700 North Charles Street  Baltimore, MD 21204 |
| South Denver Neurosurgery  7780 South Broadway, Suite 350  Littleton, CO 80122 |
| PorterCare,  Adventist Health System dba Centura Health Little Adventist Health System  2525 South Downing Street  Denver , CO 80210 |
| Kevin Brian Shrock dba  Shrock Orthopedic Research, LLC  1414 Southeast 3^rd^ Avenue  Ft. Lauderdale, FL 33316 |
| University Hospital and Medical Center  7201 North University Drive  Tamarac, FL 33321 |
| Behnam Myers, DO  3850 Sheridan Street  Hollywood, FL 33021 |
| Anthony Hall, MD  817 South University Drive, Suite 105  Plantation, FL 33324 |
| Orthopaedic Specialty Center  2400 Maryland Road, Suite 20  Willow Grove, PA 19090 |
| Abington Memorial Hospital  1200 Old York Road  Abington, PA 19001-3788 |
| East Tennessee Brain & Spine Center  701 Med Tech Parkway, Suite 300  Johnson City, TN 37604 |
| Arizona Center for Neurosurgery  3300 N. Central Avenue, Suite 2550  Phoenix, AZ 85020 |
| Surgical Specialty Hospital of AZ  6501 North 19^th^ Avenue  Phoenix , AZ 85015 |
| Ali Araghi, DO  1820 West Maryland Avenue, Suite 2  Phoenix, AZ 85015 |
| CORE Institute  14520 West Granite Valley Drive  Sun City, AZ 85375 |
| The Spine Institute of Arizona  9735 North 90^th^ Place  Scottsdale, AZ 85258 |
| The Brain and Spine Research Institute  5757 Wilshire Blvd., Suite 6  Los Angeles, CA 90036 |
| California Spine Institute  1001 Newbury Road  Newbury Park, California 91320 |
| Glendale Adventist Medical Center  1509 Wilson Terrace  Glendale, CA 91206 |
| Olympia Medical Center  5900 West Olympic Boulevard  Los Angeles, CA 90036 |
| Yale University  Yale Physicians Building  800 Howard Avenue  PO BOX 208071  New Haven, CT 06520-8047 |
| Yale University  800 Howard Avenue  New Haven, CT 06520-8071 |
| Durango Orthopedic Associates, PC  Spine Colorado  1 Mercado Street, Suite 200  Durango, CO 81301 |
| Mercy Regional Medical Center  1010 Three Springs Blvd.  Durango, CO 81301 |
| Florida Orthopaedic Associates, P.A.  740 West Plymouth Avenue  Deland, FL 32720 |
| Florida Orthopaedic Associates, P.A.  1053 Medical Center Drive, Suite 101  Orange City, FL 32763 |
| Florida Hospital Fish Memorial  1055 Saxon Boulevard  Orange City, Florida 32763 |
| Tucson Orthopaedic Institute, PC  2424 North Wyatt Drive  Tucson, AZ 85712 |
| Tucson Medical Center  5301 East Grant Road  Tucson, AZ 85712 |
| RUSH University Medical Center  University Neurosurgery  1725 West Harrison, Suite 970  Chicago, IL 60612 |
| RUSH University Medical Center  1653 West Congress Parkway  Chicago IL 60612 |
| Spine Care and Rehabilitation, Inc.  556 Eagle Rock Avenue  Roseland, NJ 07068 |
| St. Barnabas Medical Center  94 Old Short Hills Road  Livingston, NJ 07039 |
| Institute for Low Back and Neck Care  3001 Metro Drive, Suite 330  Bloomington, MN 55425 |
| Allina Health System  800 East 28^th^ Street  Minneapolis, MN 55407 |
| SUNY Stony Brook  HSC 12-80 Neurosurgery  Stony Brook, NY 11794-8122 |
| The Center for Pain Relief, Inc.  400 Court Street, Suite 100  Charleston, West Virginia 25301 |
| Neurological associates, Inc.  415 Morris Street  Charleston, WV 25301 |
| Saint Francis Hospital  333 Laidley Street  Charleston, West Virginia 25301 |
| Pacific Pain Medicine Consultants  3998 Vista Way, Suite 108  Oceanside, CA 92056 |
| Pacific Surgery Center  3998 Vista Way, Suite 106  Oceanside, CA 92056 |
| D.I.S.C Sports & Spine Center  8750 Wilshire Blvd., Suite 350  Beverly Hills, CA 90211 |
| Olympia Medical Center  5900 West Olympic Blvd.  Los Angeles, CA 90036 |
